# Maxillary Fungal Osteomyelitis Mimicking and Treated as Endodontic Pathosis

**DOI:** 10.1155/2022/1976352

**Published:** 2022-07-08

**Authors:** Sarang Suresh Hotchandani, Feroze Ali Kalhoro, Priya Rani Harjani, Mahwish Memon

**Affiliations:** Department of Operative Dentistry, Liaquat University of Medical & Health Sciences, Jamshoro, Pakistan

## Abstract

Fungal osteomyelitis is a lethal opportunistic infection that affects only a small percentage of patients present to endodontists. It is a highly contagious infection that requires urgent treatment. We discuss three cases in which endodontic pathology was initially recognized but subsequent follow-up revealed severe osteolytic fungal infection of the maxilla manifesting as acute apical abscesses in the maxillary canine region. CBCT demonstrated osteolytic abnormalities, and further histology confirmed the illness was caused by mucormycosis in patients with uncontrolled diabetes mellitus and exposure to COVID-19.

## 1. Introduction

Mucormycosis is a rare but severe fungal infection caused by a group of molds known as mucoromycetes. It is quite uncommon. Mucormycosis is more prevalent in individuals with preexisting health problems or those who take medications that decrease the immune system's ability to fight infection and disease. Diabetes patients are at an increased risk of developing mucormycosis, an invasive fungal infection that can be fatal if it spreads to other areas of the body. Individuals with hematological malignancies or who require a solid-organ transplant are at a higher risk of receiving it [1]. Mucormycosis is most frequently found in the nose, eye, and brain of those who contract it. The diagnosis may take so long due to the scarcity of symptoms. Any nonbacterial sinusitis in diabetics, even if ketoacidosis is not present, should increase the risk of mucormycosis [2].

Globally, the incidence of mucormycosis is increasing, but it is increasing at an alarming rate in India and China among people with uncontrolled diabetes mellitus. However, according to figures from the early COVID-19 times, there are 14 cases per 100,000 people in Pakistan. Cases are on the rise due to underreporting and dentists' misunderstanding of their presentation in endodontic clinics [3]. Here, three cases are reported in which patients initially complained of endodontic problems and were treated accordingly but were later diagnosed with fungal osteomyelitis masquerading as endodontic pathosis.

## 2. Case Presentations

### 2.1. Case 1

#### 2.1.1. Presenting Complaint

A 45-year-old well-educated gentleman was referred by his general dentist to the Department of Operative Dentistry and Endodontics with the complaint of persistent pain in his upper and lower anterior teeth following nonsurgical endodontic treatment of his teeth from tooth number 6 to 11 and pus discharge with swelling on his upper posterior region.

#### 2.1.2. History of Presenting Complaint and Past Treatments

The patient stated that he had been experiencing soreness in his upper and lower anterior teeth for one month and had seen his family dental practitioner. His previous dental practitioner diagnosed pulp necrosis in all his maxillary anterior teeth from tooth number 6 to 11 and initiated root canal treatment, which took approximately 20 days to complete. A few teeth remain untreated, but due to the persistence of pain, he was referred to the endodontic department for further evaluation and management. The patient had a history of dengue virus infection, which resulted in a low platelet count, and was hospitalized for many days. Apart from that, he is asymptomatic and has no significant medical history. The patient stated that he had no history of diabetes.

#### 2.1.3. Lab Workup and Results

His most recent complete blood work revealed a normal platelet count. His chemical pathology report showed C-reactive protein 3.341 mg/dl. Apart from that, he is asymptomatic and has no significant medical history. However, when glycated hemoglobin (HBA1c) was determined, the result was 6.53 percent.

#### 2.1.4. Extraoral Clinical Examination

Extraoral clinical examination revealed an obese patient with a normal gait and an excellent sense of spatial and temporal orientation. He has broad diffuse edema on the right side of his face; his temporomandibular joint testing is normal. There was no cervical lymphadenopathy.

#### 2.1.5. Intraoral Clinical Examination

**Figure 1:**
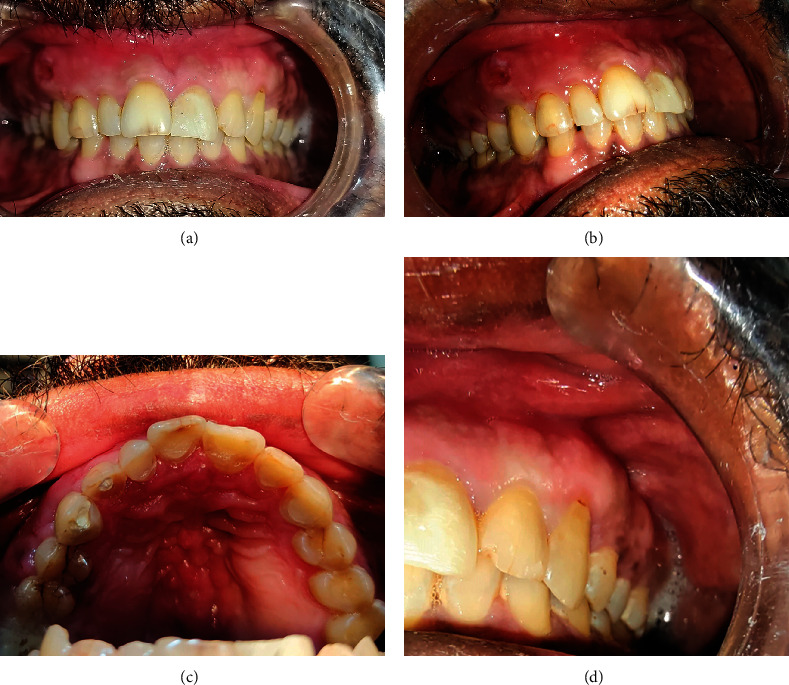
Clinical photographs of case 1: (a) frontal view, (b) right maxillary view showing the ulcer, (c) palatal view, and (d) right maxillary view showing normal gingiva.

Intraoral examination revealed multiple wear facets on the incisal edge of the maxillary anterior teeth, a supernumerary maxillary right lateral incisor, ulcer with swelling on the attached gingiva of the maxillary right canine, and widespread diffuse gingival swelling over the maxillary anterior teeth. Periodontal examination indicated grade two movement between the maxillary right third molar and the maxillary left canine, as well as grade three mobility in the maxillary right second molar, with deep probing depths. The gingiva of the maxillary right buccal region was found to be damp, spongy, and wrinkled, with pus discharge from the cervices. The palatal surface revealed a deep palate with tooth-colored restorations from tooth number 7 to 11, as well as temporary, white-colored restorations on the maxillary right supernumerary lateral incisor and tooth number 6. There were buccal and palatal swelling on the right side, with a bulbous interdental papilla between tooth number 5 and 6, and stained crack lines on the tooth number 4 and 5. The pulp tests did not result in response on the cold and electronic pulp tester (Figure 1).

#### 2.1.6. Radiographic Examination

**Figure 2:**
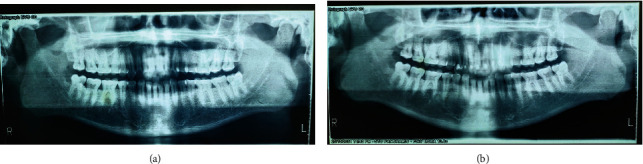
OPG of case 1 obtained at 15 days' gap.

**Figure 3:**
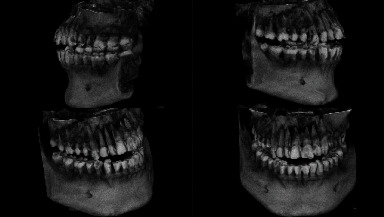
3D CBCT view of case 1 showing resorption over the maxillary alveolus region on the right side.

**Figure 4:**
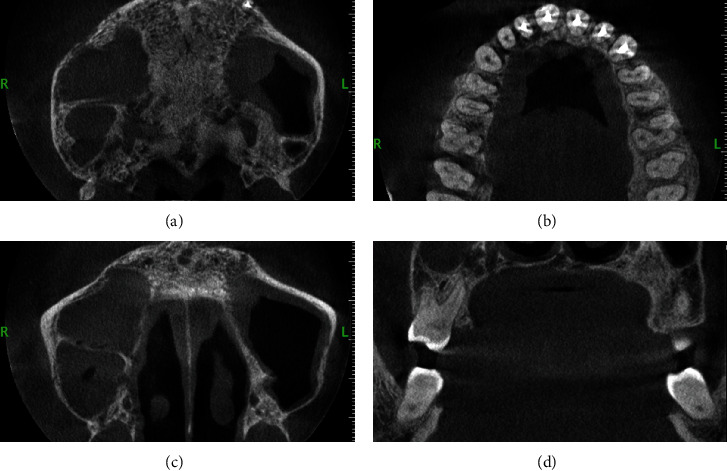
CBCT of case 1: (a–c) axial views; (d) cross-sectional view. (a) Osteolytic change in the posterior area. (b) Radiopaque obturating material in maxillary anterior teeth with resorption of alveolar process. (c) Haziness in the maxillary sinus on the right side. (d) Osteolytic changes on the palatal alveolar region on the maxillary 1^st^ molar.

Two OPGs were exposed to the patient by the referring clinician: one at the time of presentation to the general dentist and another after 20 days. The initial OPG revealed no notable results. The second OPG reveals a radiolucent line on the mesial surface of tooth number 2 and periapical radiolucency on adjacent teeth. Tooth numbers 6 and 7 exhibit obturation with restoration; however, only radiopaque restoration with empty canals is seen from tooth number 8 to 11. Multiple periapical radiographs revealed intracanal medicament in other canals that seemed empty on OPG (Figure 2). In 3D reconstruction, cone beam computed tomography (CBCT) images revealed a patchy osteolytic appearance of the maxilla from the right side of the alveolus to the left premolar area of the alveolus. Axial views revealed full haziness in the right maxillary sinus, as well as a moth-eaten aspect to the right maxilla (Figures 3 and 4).

The patient was then referred to the department of oral and maxillofacial surgery for evaluation and treatment. A computed tomography scan of the face and neck with contrast was obtained by a maxillofacial surgeon which showed mucosal thickening, resulting in partial opacification of the bilateral maxillary sinus. There was also significant erosion of the inferior wall of the maxillary sinus and the entire alveolar process of the maxilla. Additionally, a linear pathological fracture of the right maxillary alveolar process was observed along with the premolar tooth socket. The findings showed a severe maxillary sinus infection caused by a fungal infection.

#### 2.1.7. Management

The tooth number 2 was extracted, and an incisional biopsy sample was collected via intrasocket debridement. Histopathological examination was obtained. After enquiring from the oral and maxillofacial department, the patient was on the intravenous treatment of amphotericin B and was planned for future maxillectomy.

#### 2.1.8. Histopathology

Histopathological examination revealed minute fragments of extensively inflamed and ulcerated squamous mucosa, profuse granulation tissue, and necrotic bone chips. In the intrabony zones, scattered fungal hyphae were observed. Most hyphae were slender and aseptate in shape. However, the report also stated wide, ribbon-like, and aseptate hyphae. These data suggested a mixed fungal infection containing mucor.

### 2.2. Case 2

#### 2.2.1. Presenting Complaint

This 36-year-old male patient arrived for a follow-up appointment after initiating endodontic treatment in his tooth number 11 for pulp necrosis with persistent apical abscess.

#### 2.2.2. History of Presenting Complaint

This case was initiated by a postgraduate resident at the Operative Dentistry and Endodontics Department. The case was initially diagnosed as pulp necrosis with chronic apical abscess, and nonsurgical root canal therapy was planned and initiated in two visits in tooth number 11, with one visit pulpectomy followed by biomechanical preparation of canal after determining working length and intracanal calcium hydroxide placed for 15 days. However, at the follow-up visit, the patient still reported pus discharge from the same place as before. This patient was then referred to the author for additional evaluation and management. After referral, a history was obtained, and the patient claimed no major past or present medical history. The patient was prescribed by the previous resident, capsule doxycycline 100 mg for 5 days, but the pus did not cease. The patient reported COVID-19 symptoms around a month ago and is currently COVID-19 PCR-negative. He is otherwise asymptomatic and healthy with no history of diabetes.

#### 2.2.3. Clinical Examination

**Figure 5:**
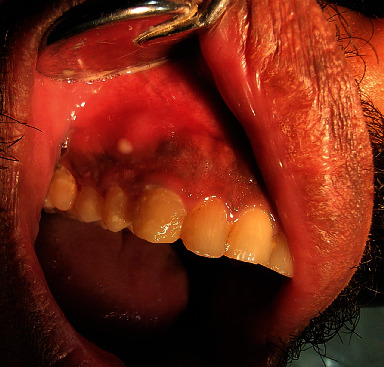
Clinical picture of case 2 showing yellowish pus discharge apical to the maxillary 1^st^ premolar.

Clinical examination revealed obvious plaque on the maxillary posterior teeth, as well as yellowish pus discharge from the attached gingiva between tooth numbers 5 and 6 (Figure 5).

#### 2.2.4. Radiographic Examination

**Figure 6:**
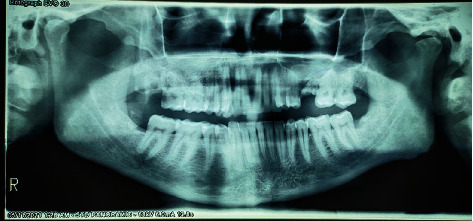
OPG of case 2.

The OPG indicated osteolytic alterations in the maxillary right posterior area and a significant cystic lesion in the maxillary left anterior region. A CBCT scan was obtained to evaluate the 3-dimensional alterations in the maxillary region (Figure 6). CBCT indicated significant osteolytic alterations in the front maxilla, as well as the involvement of the right maxillary sinus and bony sequestrum.

#### 2.2.5. Lab Reports and Results

A blood biochemical analysis revealed a normal total blood picture with elevated neutrophils and erythrocyte sedimentation rate and HBA1c of 10.9 percent.

#### 2.2.6. Management

**Figure 7:**
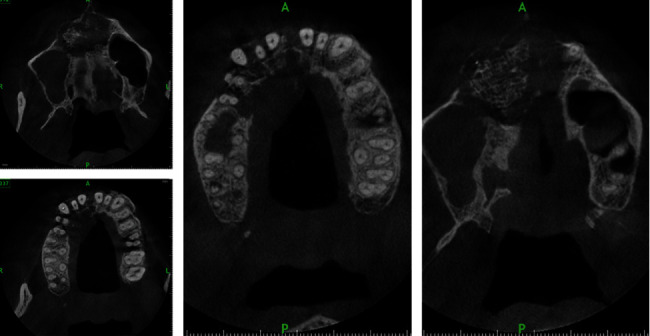
CBCT axial views of case 2 showing gross osteolytic changes with bony sequestrum with haziness on the right maxillary sinus.

**Figure 8:**
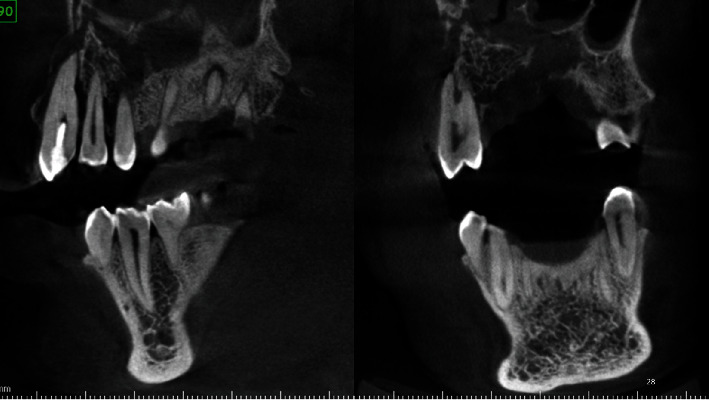
CBCT sagittal and coronal views of case 2 showing bony sequestrum with gross osteolytic changes infiltrating the floor of the maxillary sinus.

This patient was then referred to a physician for diabetes care. After a few days, the pus discharge ceased, and histology revealed that the patient had fungal osteomyelitis. The patient refused surgical therapy in favor of medicinal treatment (Figures 7 and 8).

### 2.3. Case 3

#### 2.3.1. Presenting Complaint

This 65-year-old retired officer was referred to the author for the diagnosis and treatment of persistent swelling over the gingiva in the maxillary anterior area.

#### 2.3.2. History of Presenting Complaint

This patient appeared to his family dentist with a complaint of several swellings above the tooth number 5 and 6, as well as pain while eating. After a diagnosis of pulp necrosis and symptomatic apical periodontitis, nonsurgical root canal therapy was commenced in the tooth number 6 and 7. The root canal treatment was scheduled to be completed in two visits, with pulpectomy and intracanal medicament for 15 days, calcium hydroxide. However, the pain subsided after 15 days, but the swellings did not, and another enlargement on the palatal region emerged after 7 days. The patient was subsequently referred to the author for diagnosis and care. On questioning about the patient's background, there is no history of trauma, and the patient is a regular visitor to his family dentist. He is otherwise healthy and stable right now.

#### 2.3.3. Lab Results

The patient had a history of type 2 diabetes maintained with normal laboratory tests; current whole blood HBA1c is 5.0 percent, serum creatinine 1.1 mg/dl, eGFR > 60 ml/min/1.73, normocytic normochromic blood profile, and ESR 75 mm/1Hr, and lipid profile values are normal. He sees his doctor regularly and takes his medication as prescribed.

#### 2.3.4. Clinical Examination

**Figure 9:**
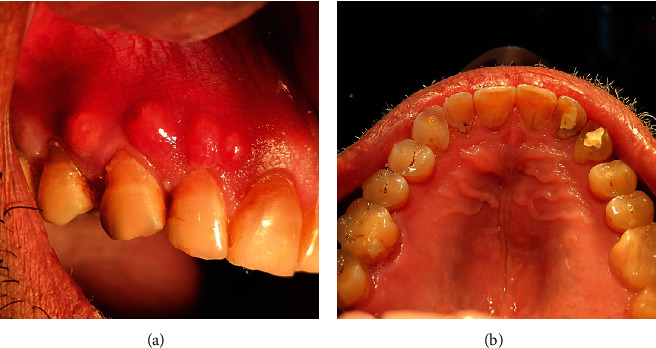
Clinical photograph of case 3 showing multiple swellings apical to the maxillary right lateral incisor and maxillary canine, with palatal swelling indicated with an arrow.

Clinical examination indicated plaque from tooth number 4 to 8, four swellings in the attached gingiva, and a cervical lesion on the tooth number 4. From the tooth number 4 through 8, there was grade 1 mobility with soft swelling in the palatal region not crossing the midline (Figure 9).

#### 2.3.5. Radiographic Examination

Periapical and OPG examinations revealed no notable abnormalities; nevertheless, CBCT was performed, which indicated osteolytic alterations in the anterior maxilla on the right side extending from the tooth number 4 through 8, as well as haziness in the left maxillary sinus.

#### 2.3.6. Management

**Figure 10:**
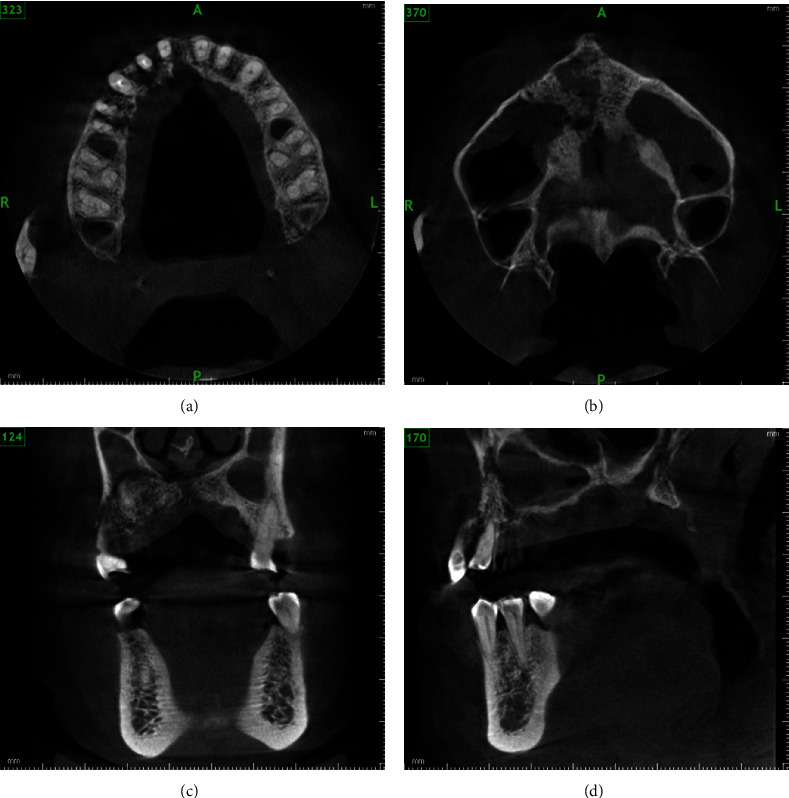
CBCT images of case 3 showing (a, b) axial views with osteolytic changes in the alveolus and palate with perforating sinus floor as seen in coronal views and sagittal views (c, d).

The patient was referred to a maxillofacial surgeon and histopathology diagnosed as fungal osteomyelitis; he is under medical treatment now (Figure 10).

## 3. Discussion

The phrase osteomyelitis comes from the Greek words osteon, myelo, and itis, which means inflammation. In 1844, Nelaton invented the term osteomyelitis. Osteomyelitis is a bone inflammation that starts in the medullary cavity and ends in the periosteum. Trauma, surgical therapy, bacteremia, fungal infection, and systemic disorders that reduce host defense mechanisms such as diabetes, cancer, anemia, radiation, malnutrition, osteoporosis, osteopetrosis, and Paget's disease are all implicated in the disease [4]. Osteomyelitis is the most frequently encountered cause of intraoral necrosis (maxillary necrosis). Maxillary necrosis is a condition that can be caused by bacterial osteomyelitis, herpes zoster, trauma, surgical infections, or fungal infections such as mucormycosis and aspergillosis. Mucormycosis is a fungal infection of the nasal cavity and paranasal sinuses caused by Mucorales fungus. It is disseminated via breathed fungus spores, swallowed fungus spores, or infected mucosa or skin. It is highly contagious and has the potential to be lethal in immunocompromised, handicapped, or diabetic patients [5, 6].

Candida osteomyelitis is a condition that can be exacerbated by diabetes. If fungus is not identified and treated promptly, it can cause wound healing to be impaired [7]. Diabetic patients are more likely to contract uncommon infections and have slower healing wounds. Macrophages' chemotaxis, phagocytosis, and cytokine release are all affected by diabetes' chronic hyperglycemic condition, which alters the cell-mediated immune system. Furthermore, in patients with diabetic ketoacidosis (DKA), phagocyte dysfunction results from reduced intracellular oxidative and nonoxidative killing of pathogens [8, 9]. In our cases, two patients had no history of diabetes before the authors seeking a lab report that revealed the presence of undetected uncontrolled type 2 diabetes mellitus, and one patient had stable diabetes. This is consistent with Ankita's [6] study, which found that uncontrolled hyperglycemia was a factor in 40% of instances of fungal osteomyelitis of the maxilla.

COVID-19 causes immunological dysregulation, which increases the likelihood of opportunistic fungal infection. It can also cause cytokine bursts in the body, resulting in vascular damage and reduced blood flow, which results in bone necrosis [10]. Two of our three patients had a history of diagnosed COVID-19, while the third had a probable but unconfirmed diagnosis. It is possible that glucocorticoid medication results in insulin resistance and diabetes, as well as dysregulation of the vascular system, in COVID-19. Both COVID-19 and diabetes have been linked to an increased risk of mucormycosis.

A wide range of imaging modalities may be required to accurately identify osteomyelitis in some cases. First, conventional radiography should be utilized since it provides a complete picture of the bone and soft tissue architecture and disease of the region. Because of the low sensitivity and specificity of plain radiography for the early diagnosis of acute osteomyelitis [11], our patient's OPGs and periapical radiographs returned no meaningful results. Up to 80% of patients will have a normal radiograph in the first two weeks following the start of the infection. Plain films cannot reveal the earliest indications of bone marrow edema [12]. Positron emission tomography (PET), single-photon emission computed tomography (SPECT), and magnetic resonance imaging are all good ways to find osteomyelitis. None of the tests had an advantage over the other in terms of how accurate they were at diagnosing people. It is easier for people to get an MRI because it is more common and does not cause them to be exposed to dangerous ionizing radiation. However, we used cone beam computerized tomography (CBCT), which is used a lot in dental work, to see what was going on. After we started the endodontic treatment, we used CBCT to find out what was causing the sinus drainage even though the endodontic treatment was good.

The patients were referred to an oral and maxillofacial surgeon for histopathology for confirmation and subsequent management due to the multidisciplinary nature of mucormycosis treatment, which includes the use of antifungal medications, surgical debridement, and management of underlying pathology that may contribute to the development of the fungal infection. The first line of therapy for this illness is liposomal amphotericin B. On the other hand, medical therapy alone will not cure the problem, and rigorous surgical treatment will eventually be necessary to augment the efficacy of antifungal drugs.

When detected early, this fungal infection can be treated medically with minor surgical debridement. Patients with a history of diabetes and COVID-19 should be screened with CBCT if they present with an intraoral lesion that looks like a periapical abscess due to the limited sensitivity of conventional routine radiographs used in endodontics to diagnose fungal osteomyelitis.

## 4. Conclusions

In the case of a diabetic patient with pulp necrosis and apical pathosis without potential localized cause in the maxillary canine and premolar region and a history of suspected or confirmatory COVID-19 infection, cone beam computed tomography should be performed to rule out osteomyelitis to avoid delayed treatment of this highly aggressive illness.

## Data Availability

The data that support the findings of this study are available from the corresponding author upon reasonable request.
